# Selected protein expression in a new prognostic model for patients with non-muscle-invasive bladder cancer

**DOI:** 10.1007/s00432-020-03202-0

**Published:** 2020-04-01

**Authors:** Aleksandra Semeniuk-Wojtaś, Arkadiusz Lubas, Szczepan Cierniak, Urszula Brzóskowska, Tomasz Syryło, Henryk Zieliński, Rafał Stec

**Affiliations:** 1 Oncology Department, Military Institute of Medicine, 128 Szaserów St., 04-141 Warsaw, Poland; 2 Internal Diseases, Nephrology and Dialysis Department, Military Institute of Medicine, 128 Szaserów St., 04-141 Warsaw, Poland; 3 Patomorphology Department, Military Institute of Medicine, 128 Szaserów St., 04-141 Warsaw, Poland; 4 General, Functional and Oncological Urology Department, Military Institute of Medicine, 128 Szaserów St., 04-141 Warsaw, Poland; 5 Oncology Department, Medical University of Warsaw, 19/25 Stępińska St., 00-739 Warsaw, Poland

**Keywords:** Bladder cancer, NMIBC, Ki-67, p53, Surviving, RECINT model

## Abstract

**Introduction:**

After transurethral resection of a bladder tumor, patients frequently have a recurrence of the disease, thereby requiring adjuvant therapy.

**Purpose:**

The study aimed to determine the prognostic value of expression levels of p53, Ki-67, and survivin, and to develop a new prognostic model for patients with non-muscle-invasive bladder cancer (NMIBC) after transurethral resection of a bladder tumor.

**Methods:**

The study group consisted of 101 patients with primary NMIBC. Univariate followed by multivariate Cox proportional hazard regression analysis was performed to obtain a model including the smallest possible number of descriptive variables with the highest statistical significance and impact on risk.

**Results:**

The RECINT model (RECurrence In Not Treated) including factors independently associated with cancer recurrence (tumor size [HR 1.148; *p* = 0.034], intensity of the color reaction for p53 [HR 1.716; *p* = 0.008], Ki-67 [HR 3.001; *p* = 0.022], and survivin [HR 1.461; *p* = 0.021]) adequately stratified recurrence free-survival (*R*^2^ = 0.341, p < 0.001) in patients with primary NMIBC. Patients with the lowest RECINT score (0–6) had the lowest probability of cancer recurrence (1- and 5-year recurrence of 16%) in comparison with other groups (*p* < 0.001).

**Conclusions:**

The RECINT model may be useful for stratifying the risk of recurrence in patients with non-muscle-invasive bladder cancer and may allow for identification of those who may benefit the most from adjuvant BCG immunotherapy.

## Introduction

Bladder cancer is one of the most common malignant tumors in the world (Ferlay et al. 2012). In about 75% of patients, the cancer is diagnosed at the mucosal (Ta, Tis) or submucosal (T1) stage (Burger et al. 2013). The basic treatment for non-muscle-invasive bladder cancer (NMIBC) is transurethral resection of the bladder tumor (TURBT), which allows effective removal of the tumor. However, observational studies indicate that 48%–61% of patients have a recurrence of the disease, thereby requiring adjuvant therapy and close monitoring for many years (Alsheikh et al. 2001; Sylvester et al. 2006; Zieger et al. 2000). The method that most effectively reduces the risk of recurrence is intravesical BCG immunotherapy, which is, however, associated with significant adverse effects (Babjuk et al. 2010; Malmström et al. 2009; Shang et al. 2011). The most frequently reported adverse effects of BCG immunotherapy are chemical and bacterial cystitis, hematuria, malaise, fever, and urinary incontinence. The literature also describes sepsis as a complication of BCG (Brausi et al. 2014). Poletajew et al. also found frequent deviations from the recommended BCG regimen in the adjuvant treatment of bladder cancer, including an inadequate duration of therapy (Poletajew et al. 2017).

Analyses performed by many researchers have allowed the development of prognostic models to assess the short- and long-term risks of tumor recurrence. Among them, the most important are tables developed by the European Organization for Research and Treatment of Cancer (EORTC) and Club Urológico Español de Tratamiento Oncológico (CUETO). The EORTC has developed a prognostic model based on the analysis of the course of disease in patients with bladder cancer in the Ta or T1 stage. Seventy-eight percent of patients enrolled in the study received adjuvant treatment—the majority of patients were treated with doxorubicin, cisplatin, mitomycin, or epirubicin (less than 10% of patients underwent intravesical BCG immunotherapy). Statistical analyses were performed by the researchers to determine the risk factors for recurrence, including the number and size of neoplastic lesions, presence of concomitant carcinoma in situ (CIS), T-stage according to the TNM classification, histological grade of cancer, and previous recurrence rate (Sylvester et al. 2006).

The CUETO scoring model is based on the analysis of the disease course in patients receiving intravesical BCG therapy. Analyses carried out by researchers have shown that the parameters associated with an increased risk of tumor recurrence in this group of patients are sex, age, number of neoplastic lesions, previous tumor recurrences, T-stage according to the TNM classification, presence of coexisting CIS, and histopathological grade of the tumor (Fernandez-Gomez et al. 2009). The choice of the optimal treatment regimen can also be facilitated by assigning patients to one of the three risk groups according to the criteria developed by the European Association of Urology (EAU) (Babjuk et al. 2017). The EAU risk groups were determined on the basis of information available in the literature, in particular, data from the EORTC risk tables.

However, the use of the above-mentioned algorithms in patients after primary tumor resection is questionable. A factor that limits the use of these models is the fact that patients included in the analysis received different schedules of adjuvant therapy, which could have influenced the number of recurrences and the time to recurrence (Kurth et al. 1997; Oosterlinck et al. 1993). Moreover, in the available models, the number of recurrences per year has high prognostic value, and this parameter cannot be assessed in patients who have been diagnosed with cancer for the first time. These factors provide a rationale for developing a new prognostic model that would allow more precise stratification of the risk of recurrence in patients with primary bladder cancer and identification of a group of patients who would benefit significantly from intravesical BCG immunotherapy.

The study aimed to develop a new prognostic model for patients with primary non-muscle-invasive bladder cancer after transurethral resection of the bladder tumor.

## Materials and methods

### Materials

For this retrospective study, clinical data from patients’ medical history and paraffin-embedded sections of papillary urothelial carcinoma from the Department of General, Functional, and Oncological Urology of the Military Institute of Medicine in Warsaw removed during transurethral resection of the bladder tumor between 2010 and 2015 were used. The study was approved by the Bioethics Committee of the Military Institute of Medicine in Warsaw (No. 477).

We included all patients with primary bladder cancer who did not receive adjuvant BCG immunotherapy (e.g., due to lack of consent to treatment, comorbidities, or lack of indications in the opinion of the treating oncologist). Patients whose follow-up period was shorter than 12 months and those with tumor recurrence within the upper urinary tract were excluded from the study.

Patients who enrolled in the study were subjected to control cystoscopy. Second TURB had been performed in selected patients 2 to 4 weeks after initial resection. In those in whom neoplastic lesions were detected during the cystoscopy, TURBT was performed, and the removed tissues were transferred for histopathological examination. Histopathologically-confirmed bladder cancer detected during the second TURB was interpreted as an incomplete resection and was not assessed as a recurrence. Each subsequent case of histopathologically-proven urothelial carcinoma detected during control cystoscopy was considered as a recurrence.

### Immunohistochemistry

The hematoxylin and eosin (HE) stained tissue blocks were retrieved and reevaluated by a pathologist, and the representative areas corresponding to tumor classification and grading were selected. Tumor histological differentiation was graded according to the 1973 WHO classification, and the assessment of the clinical stage of cancer was based on the criteria of the seventh edition of tumor-node-metastasis (TNM) classification by the International Union Against Cancer (UICC, Union Internationale Contre le Cancer) (Mostofi et al. 1973; Wysocka et al. 2010). The unknown “T’ feature was demonstrated mainly due to extensive thermo-coagulation damage. Sometimes the tumor was removed superficially (only the epithelial layer), and only small fragments of the normal smooth muscle lacking epithelial lining in the tumor bottom margin were found.

Immunohistochemistry was performed on 3-μm-thick tissue sections. Monoclonal mouse antibodies (DAKO) were used to evaluate the expression of the proteins of interest (p53—clone D0-7; Ki-67—clone MIB1; survivin—clone 12C4). For microscopic assessment of the color reaction with antibodies against survivin, p53, and Ki-67, the intensity of nuclear staining was scored on a 4-degree intensity score (IS) scale (Allred et al. 1998). The results obtained were classified as follows: the highest score (3 +) was defined as strong staining of the cell membrane. The reaction was scored as (2 +) when there was moderate staining. A score of (1 +) indicated weak nuclear staining. A result of (0) indicated a complete lack of expression. Positive staining was defined as obtaining a color reaction of any intensity.

### Statistical analysis

Statistical analysis of the results was performed using the Statistica software (StatSoft Inc.), version 12. The result of the statistical test was considered significant if the test probability p was lower than the type I error value of 0.05.

### Prognostic model development

To assess the association of potential variables affecting recurrence-free survival (variables whose impact on recurrence-free survival has been proven in other studies and new variables derived from the immunohistochemical study in the analyzed population), an analysis was performed using the univariate Cox proportional hazards regression model. Variables having a significant impact on recurrence-free survival were used to build a Cox proportional hazards regression model. Among the collinear variables describing the same feature, only those with the highest significance were included in the model. Using multivariable regression analysis, we developed the model with the highest degree of matching that simultaneously contains the smallest possible number of variables. In the selected prognostic model, each variable was assigned a score within the range corresponding to the individual hazard ratio (HR) and weight-of-evidence (WoE) coding based on information about the predictive power of specific feature variants regarding the risk of recurrence. For each case, the total score was calculated as a result of the model by summing the scores for individual variables. To adequately stratify the risk of recurrence in the study population, the scores obtained using this model were categorized on the basis of WoE results into four groups defined by a range of points. Differences between groups within the model were assessed using the log rank test or chi square test (*χ*^2^).

## Results

### Clinicopathological characteristics of the study population

The study group consisted of 101 patients (87 men and 14 women) with primary papillary bladder cancer. Detailed characteristics of the patient group are presented in Table 1.

**Table 1:** Patient characteristics

Feature	Number of patients (*n*)	Percentage (%)
Gender		
Male	87	86.14
Female	14	13.86
Stage		
Tis	7	6.93
Ta	39	38.61
T1	13	12.88
Not known	42	41.58
Concomitant Tis		
Yes	2	1.98
No	56	55.45
Not known	43	42.57
Grade		
G1	50	49.51
G2	45	44.55
G3	5	4.95
Not known	1	0.99
Number of tumors		
1	67	66.34
2–3	18	17.82
≥ 4	16	15.84
Diameter of the largest tumor		
< 1 cm	4	3.96
1–2.5 cm	45	44.55
≥ 3 cm	45	44.55
Not known	7	6.94
Recurrence		
Yes	66	65.35
No	35	34.65

The overall median follow-up period was 13 (range 2–87) months. During the analyzed period, neoplastic disease recurred in 66 patients, which constituted 65.35% of the examined group. The median time to recurrence was 8 (range 2–60) months.

### Immunohistochemical assessment of protein expression levels

**Fig. 1:**
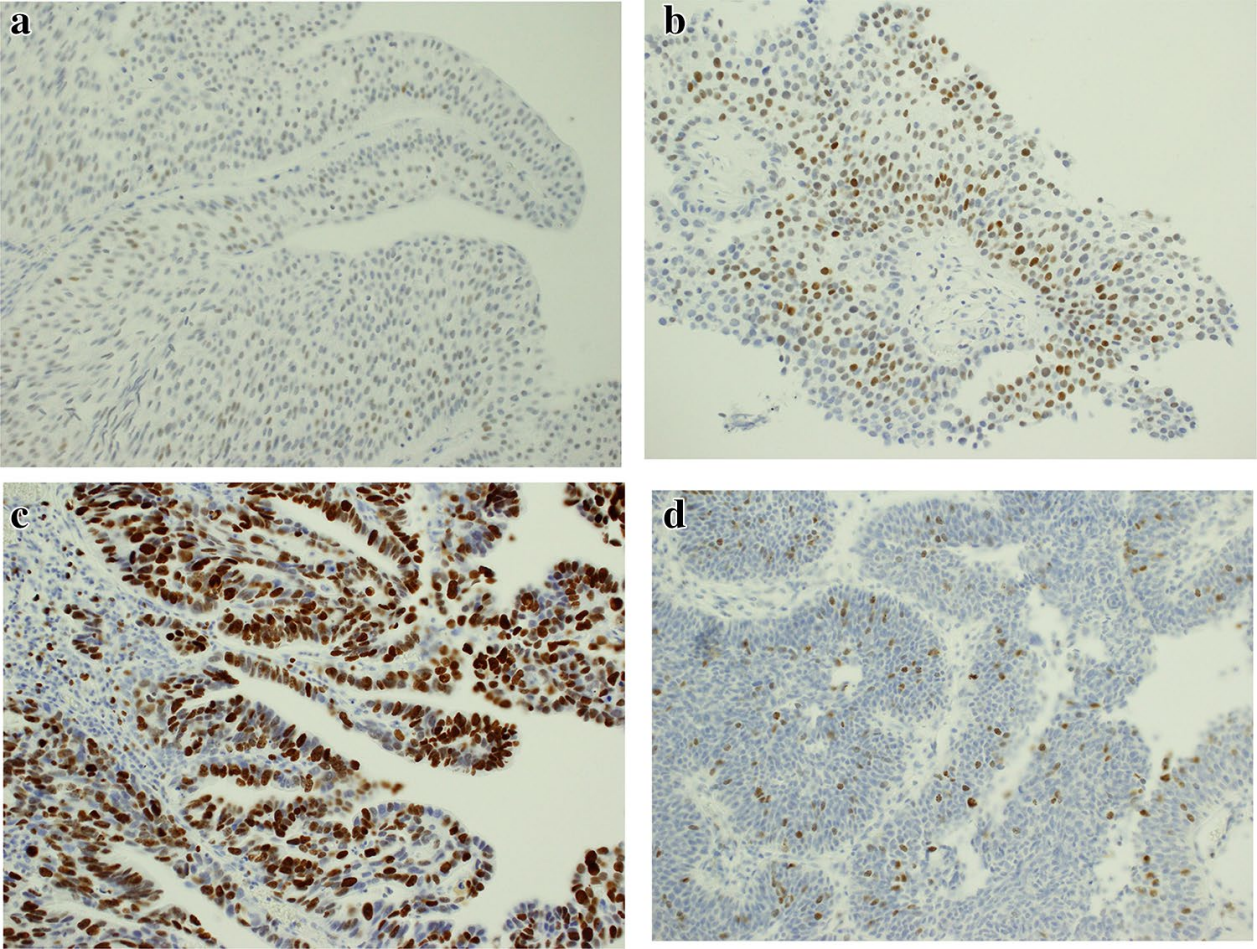
**a** Bladder cancer—an example of positive staining with anti-p53 antibodies (× 200); IS1. **b** Bladder cancer—an example of positive staining with anti-p53 antibodies (× 200); IS2. **c** Bladder cancer—an example of positive staining with anti-Ki-67 antibodies (× 200); IS3. **d** Bladder cancer—an example of positive staining with anti-survivin antibodies (× 200); IS2

Immunohistochemistry was performed on sections of primary papillary bladder cancer removed during transurethral resection of the tumor. Expression of the p53 protein was confirmed in all of the examined tissues. Nuclear staining intensity was low in 14 (13.86%) patients, moderate in 61 (60.4%) patients, and high in 26 (24.75%) patients. Expression of Ki-67 protein was demonstrated in all analyzed cases. The intensity of the color reaction was low in 5 (4.95%) cases, moderate in 6 (5.94%) cases, and high in the other 90 (89.11%) cases. Survivin expression was found in 100 (99%) cases. Nuclear staining intensity was low in 45 (43.69%) patients, moderate in 43 (41.75%) patients, and high in the remaining 12 (11.65%) patients. Representative images of cases and histochemical staining are shown in Fig. 1. The immunostaining results depending on the different tumor subclassifications are presented in Table 2.

**Table 2:** Immunostaining results depending on the different tumor subclassifications

	p53			Survivin			Ki-67		
	IS 1 [%]	IS 2 [%]	IS 3 [%]	IS 1 [%]	IS 2 [%]	IS 3 [%]	IS 1 [%]	IS 2 [%]	IS 3 [%]
TisG1	12.5	7.3	4.55	16	3.03	0	0	0	7.35
TisG2	12.5	2.44	4.55	4	3.03	10	0	0	4.41
TisG3	0	0	0	0	0	0	0	0	0
TaG1	62.5	26.83	9.09	24	21.21	20	50	33.3	23.53
TaG2	0	31.7	36.36	36	27.27	20	50	0	29.41
TaG3	0	0	4.55	0	3.03	10	0	33.3	1.47
T1G1	0	2.44	4.55	0	0	10	0	0	2.94
T1G2	12.5	24.4	13.64	20	21.21	20	0	0	20.59
T1G3	0	4.88	22.73	0	21.21	10	0	33.3	10.29

*IS* intensity score, [%] percentage

### Evaluation of the prognostic value of p53, Ki-67, and survivin expression in non*-*muscle-invasive bladder cancer

The grade, tumor size, and expression level of p53, Ki-67, and survivin determined by the intensity of nuclear staining were independently associated with the risk of recurrence of bladder cancer in univariate Cox proportional hazard regression analysis of recurrence-free survival (Table 3).

**Table 3:** Results of univariate Cox proportional hazard regression analysis of recurrence-free survival

	HR	95% confidence interval	*p*
Grade	1.824	1.226–2.715	0.003
T Tcis, Ta, T1	1.359	0.756–2.441	0.304
Number of lesions	1.074	0.992–1.163	0.075
The tumor size	1.179	1.04–1.337	0.009
p53 IS	1.694	1.146–2.504	0.008
Survivin IS	1.432	1.054–1.947	0.021
Ki-67 IS	3.227	1.181–8.813	0.022

*IS* intensity score, *HR* hazard ratio, *p* statistical significance level

The features determined by EORTC and EAU were evaluated in the analyzed population in a multivariable Cox proportional hazards regression model (Tables 4 and 5). A statistically significant independent association was found only between recurrence-free survival and the number of tumors in the EORTC model (*R*^2^ = 0.213, AIC = 516.5) (Table 4). However, none of the factors were statistically significant in the EAU model (Table 5).

**Table 4:** Cox proportional hazards regression analysis model including EORTC risk factors

	HR	95% confidence interval	*p*
Tumor recurrence	2.065	00.654–6.524	0.216
Concomitant CIS	2.125	0.487–9.272	0.315
Grade	1.360	0.882–2.099	0.163
T category (1, 2–3, ≥ 4)	1.098	0.647–1.864	0.728
Number of tumors	1.532	1.013–2.316	0.042
Tumor size (< 3, ≥ 3)	1.638	0.977–2.748	0.061

*CIS* carcinoma in situ, *G* grade, *p* statistical significance level, *HR* hazard ratio

**Table 5:** Cox proportional hazards regression analysis model including EAU risk factors

	HR	95% confidence interval	*p*
Concomitant CIS	2.239	0.472–10.609	0.309
Tumor recurrence	2.302	0.723–7.328	0.158
Number of tumors (1, 2, ≥ 3)	1.644	0.727–3.718	0.232
Tumor size (< 3, ≥ 3)	1.325	0.887–1.980	0.169
Grade	1.361	0.98–1.891	0.065

*CIS* carcinoma in situ, *p* statistical significance level, *HR* hazard ratio

**Fig. 2:**
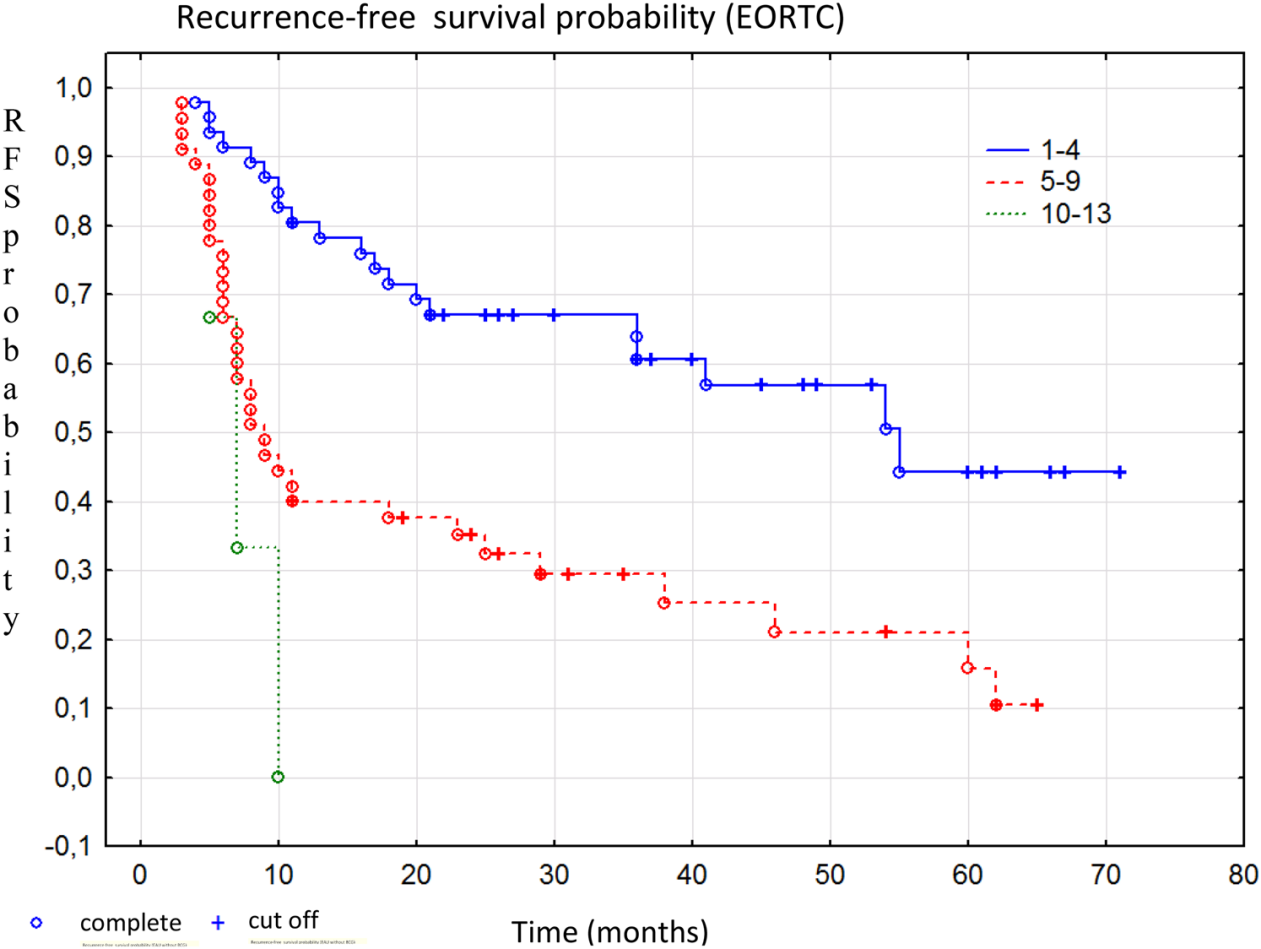
Kaplan–Meier curves of recurrence-free survival by risk groups according to the EORTC model, Chi square *p* < 0.001

**Fig. 3:**
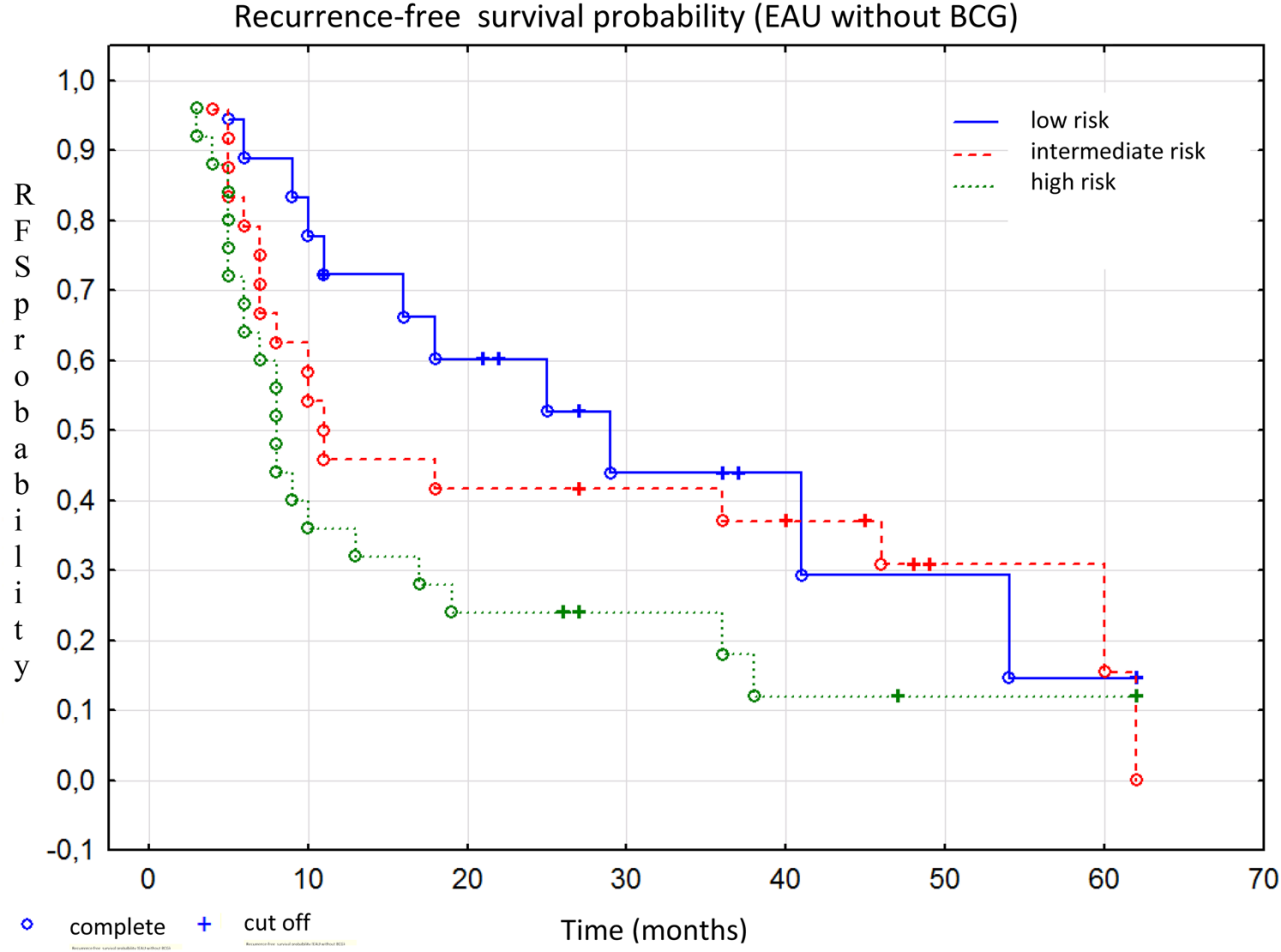
Kaplan–Meier curves of recurrence-free survival by risk groups according to the EAU risk tables, Chi square *p* = 0.026

Kaplan–Meyer curves by recurrence risk category according to the EORTC model are presented in Fig. 2. The risk of tumor recurrence in the analyzed group of patients according to the EAU risk tables is presented in Fig. 3.

### Prognosis of the risk of bladder cancer recurrence

**Fig. 4:**
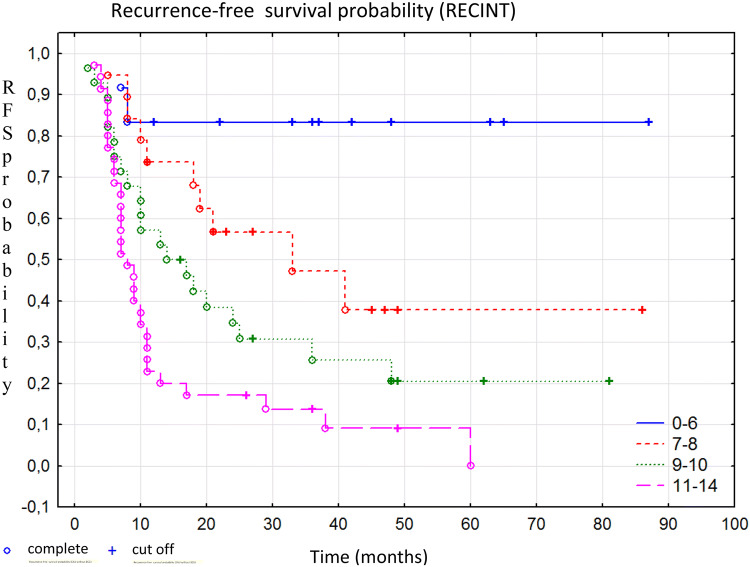
Kaplan–Meier curves of recurrence-free survival by risk groups according to the RECINT model, Chi square *p* < 0.001

To identify the group of patients with the highest risk of recurrence, who could benefit the most from adjuvant BCG immunotherapy, the RECINT (RECurrence In Not Treated) model was created. The model was generated using clinicopathological parameters that showed a statistically significant correlation with disease recurrence. The model that was most strongly associated with recurrence-free survival (*R*^2^ = 0.341, AIC = 500.918, *p* < 0.001) included tumor size (defined as diameter ≤ 2 cm, 2–4 cm or > 4 cm), the intensity of the color reaction for p53 determined by the IS (range 0–3), and the intensity of the nuclear reaction for Ki-67 (category 1: IS = 0–2, or 2: IS = 3), and survivin (category 1: IS = 0–1, or 2: IS = 2–3). In the multivariate Cox proportional hazards regression analysis, the G feature was not significant and was excluded from the model. The results of the multivariate analysis are presented in Table 6. Specific parameters are assigned scores based on HR, as shown in Table 7. The total score for the lowest risk of recurrence was equal to 0. Patients with a total score of 14 points had the highest probability of recurrence. Based on the total score obtained in the model, the patients were divided into four groups with different levels of recurrence risk. The first group included patients whose score was within the range of 0–6 points; the second group, 7–8 points; the third group, 9–10 points; and the fourth group, 11–14 points. Kaplan–Meyer curves by recurrence risk categories according to the RECINT model are presented in Fig. 4. The probability of recurrence-free survival at 1, 3, and 5 years following primary tumor resection is shown in Table 8.

**Table 6:** Results of Cox proportional hazards regression analysis for recurrence-free survival (RECINT model)

	HR	95% confidence interval	*p*
IS-Ki-67	3.001	1.165–7.730	0.022
IS-Survivin	1.461	1.057–2.019	0.021
IS-p53	1.716	1.146–2.571	0.008
The tumor size	1.148	1.01–1.306	0.034

*IS* intensity score, *p* statistical significance level, *HR* hazard ratio

**Table 7:** Scores of selected parameters according to the RECINT model

	Score
Tumor size	
0–2 cm	0
2–4 cm	1
> 4 cm	2
Ki-67 IS	
0–2	0
3	6
p53 IS	
0–1	0
2	2
3	4
Survivin IS	
0–1	0
2–3	2
Maximum score	14

**Table 8:** Probability of recurrence-free survival according to the RECINT model

Group	Follow-up period [years]	RFS [%]	HR
1	1	84.0	0.17
	2	84.0	0.17
	3	84.0	0.17
	5	84.0	0.17
2	1	74.6	0.29
	2	58.0	0.54
	3	49.1	0.71
	5	40.2	0.91
3	1	55.2	0.59
	2	36.3	1.01
	3	27.6	1.29
	5	22.6	1.49
4	1	27.0	1.31
	2	16.8	1.78
	3	12.0	2.11
	5	4.4	3.11

*RFS* recurrence-free survival, *HR* cumulative hazard ratio for recurrence

## Discussion

Limitations of the available tools used to determine the probability of recurrence, problems associated with immunotherapy, and the results of studies indicating the prognostic value of new molecular markers justify the attempts to improve the available prognostic models.

The model developed by the EORTC is recognized as one of the main prognostic tools in NMIBC patients, so it was used in our study to determine the probability of recurrence of bladder cancer in the analyzed population. In our study, we excluded patients with a follow-up period of less than 12 months to ensure that the recurrence is not due to an insufficiently removed primary tumor. The unknown “T’ feature was demonstrated mainly due to extensive thermo-coagulation damage. Sometimes the tumor was superficially removed (only the epithelial layer) and only small fragments of the normal smooth muscle lacking epithelial lining in the tumor bottom margin were found. The study was based on the course of the disease in patients who were not qualified for BCG therapy after the primary tumor resection. This allowed the isolation of a group of patients who absolutely require BCG therapy and will obtain the greatest benefit from it, which is particularly important due to the possible side effects associated with this method of treatment and difficulties in the availability of BCG.

In our population, the majority of the variables determined by the EORTC did not show statistically significant associations with recurrence. Moreover, in the study group, no patients scored 0 points according to the EORTC scoring system, so the analyzed group of patients was divided into three subgroups. The risk of recurrence in the group of patients who scored 1–4 points (subgroup of patients with the lowest risk of recurrence) within two years following the primary tumor resection was over 30% and was comparable to the reported likelihood of recurrence in the group of patients receiving adjuvant BCG immunotherapy (Nadler et al. 1994; Nepple et al. 2010), so the use of intravesical BCG immunotherapy in each patient is justified; however, the qualification of all patients for adjuvant intravesical BCG therapy is not realizable because of the high toxicity and limited availability of immunotherapy. Similar results were obtained when we analyzed the risk of recurrence according to EAU tables: the appointed variables were not statistically significant in the analyzed group of patients.

The results of the analysis show that the EORTC and EAU models are not optimal instruments for assessing the risk of recurrence. The use of the EORTC tables in the group of patients after resection of the primary tumor is questionable, mainly because of the high value of the number of recurrences that occurred during the first year of follow-up. Furthermore, our patients differ from the EORTC population because, as we mentioned above, the EORTC tables were constructed with data on patients with primary and secondary bladder tumors, and most of these patients received adjuvant treatment, whereas our group was composed only of patients with primary bladder cancer without adjuvant treatment.

Attempts have been made by many researchers to improve the algorithms developed by the EORTC. Some authors have focused on the preparation of prognostic models for specific groups of patients, while others have attempted to determine the probability of tumor recurrence by evaluating gene polymorphisms, inflammation, or the presence of tumor markers in urine (Ieda et al. 2016; Kim et al. 2016; Maturana et al. 2016; Chen et al. 2012; Cui et al. 2017; Todenhöfer et al. 2014; Lammers et al. 2016). The possibility of improving the accuracy of these algorithms by means of immunohistochemistry was also analyzed (Ding et al. 2014; Passoni et al. 2016). Passoni et al. supplemented the algorithms developed by the EORTC and CUETO with the levels of expression of p53, p21, p27, Ki-67, and cyclin E proteins, showing that the inclusion of Ki-67 expression slightly increases the prognostic ability of the models (Passoni et al. 2016).

In this study, we analyzed the expression levels of the proliferation marker Ki-67 and two markers of apoptosis, p53 and survivin, because the cell cycle regulation pathway is one of the most common dysregulated pathways in bladder cancer (Weinstein JN Cancer Genome Atlas Research Network 2014). In addition, these proteins are simple to analyze, so tests can be performed in all laboratories.

The expression of Ki-67 is detected in all proliferating cells except for cells in the G0 phase, and the maximum level of the protein is found in the G2 phase or during mitosis. The function of Ki-67 protein probably relies on heterochromatin organization and prevention of the aggregation of mitotic chromosomes, which leads to their appropriate distribution in daughter cells (Sobecki et al. 2016). The Ki-67 protein is a marker of the growth fraction of cells and, therefore, can also be recognized as a marker of the biological aggressiveness of cancer.

The p53 protein can induce apoptotic cell death and is expressed, inter alia, in response to DNA damage. In normal cells, p53 regulates the expression of its downstream target genes to regulate a wide variety of biological processes and prevent tumorigenesis (Aubrey et al. 2018). Mutations in TP53 are the most common mutations in muscle-invasive bladder cancer (MIBC) and were detected in 49% of the samples and in 58% of high-grade (T1G3) non-muscle-invasive bladder cancers (NMIBC) (Weinstein JN Cancer Genome Atlas Research Network 2014; Lopez-Knowles et al. 2006).

Survivin is also a protein expressed during the cell cycle, mainly at the G2/M phase, and has a dual function in cells. It has been shown that survivin can inhibit apoptosis through caspase-dependent and independent pathways and can take part in the activation of cell death pathways. Survivin may be a target of p53 for its action, and p53 function may be regulated by survivin activity (Jaiswal et al. 2015). Apollo et al. detected a negative correlation between survivin expression and p53 mutational status in NMIBC (Apollo et al. 2019).

In our study, statistical analysis based on the results of immunohistochemical assessment and clinical data in patients with primary bladder cancer without adjuvant intravesical treatment enabled the development of a new model that would more accurately determine the probability of a recurrence. The RECINT model presented in this paper is based on the evaluation of four parameters and includes tumor diameter and the intensity of nuclear staining for p53, Ki-67, and survivin. Selected parameters were assigned scores reflecting their proportional contribution to the risk of cancer recurrence. Based on the total score obtained in the model, the patients were divided into four groups with different levels of recurrence risk. Patients in the first group showed the lowest risk of recurrence. Their total scores were 0 and 6 points, which means that the tumor removed during TURBT was small and characterized by a low level of expression of proteins regulating the cell-division cycle. In this group of patients, the probability of recurrence within 1 year after tumor resection was 16% and remained unchanged in the subsequent years of follow-up. The risk of recurrence in the first group according to the RECINT model was also lower than in the group of patients who received BCG immunotherapy (20% at 1-year and about 30% at 5-year follow-up). In the patients classified in the second group according to the RECINT model (7–8 points), the risk of recurrence was 25% at 1-year follow-up and 42% at 2-year follow-up and was similar to the recurrence risk in the BCG-treated group reported in the literature. However, in subsequent years, the probability of recurrence in patients in the second group gradually increased, reaching 60% at 5 years. Patients in the third group (9–10 points) were characterized by a high, 50% risk of recurrence within the first year of follow-up. The highest risk of recurrence was observed in patients classified in the fourth group, who had large tumors of diameter > 4 cm composed of cells with high division activity (11–14 points). The recurrence risk in this group was 73% within the first year and over 95% within the five years following the resection of the primary tumor. The above results indicate that patients classified in the first group according to the RECINT model may not benefit significantly from the use of intravesical adjuvant BCG immunotherapy. However, BCG therapy should be considered in the remaining patients, especially in patients from the third and fourth groups, who were at high risk of recurrence.

In the presented study, as in the study of Passoni et al., the prognostic value of proteins determining the tumor oncological potential was assessed, but the results obtained were different (Passoni et al. 2016). It was shown that the expression levels of proteins involved in the regulation of the cell-division cycle categorized using the intensity of the obtained color reaction have a higher prognostic value than the "classic" histopathological prognostic factors, which include the extent of bladder wall invasion by the tumor and histological grade of the tumor. Differences in the results obtained may have been caused by several factors. Only patients with primary bladder cancer who were not subjected to BCG immunotherapy were included in the present study, while the population of patients analyzed by Passoni et al. was heterogeneous: it included patients with primary and recurrent bladder cancer, and 33% of the study population represented patients who had received adjuvant intravesical BCG immunotherapy. The methods of evaluating protein expression levels were also different: in the study of Passoni et al., the obtained color reactions were categorized according to the percentage of stained cell nuclei and in our study, it’s according to the staining intensity. A discrepancy between the obtained results may also be caused by differences in the assessment of tumor stage by pathomorphologists despite defined diagnostic criteria for transitional cell carcinoma (in one study aimed at verification of histopathological classification, 20% of diagnoses of cancer identified as T1 were changed, while in another study, initial diagnoses were confirmed only in 50%–56% of the cases) (Passoni et al. 2016; Weinstein JN Cancer Genome Atlas Research Network 2014). Results similar to our findings were obtained by Ding et al. The researchers showed that considering the level of Ki-67 protein expression when determining the risk of recurrence using the EORTC model improves the accuracy of the diagnostic process (Ding et al. 2014).

An advantage of the proposed RECINT model based on factors related to the course of the disease in patients after TURBT is the classification of patients who have not undergone BCG therapy after resection of the primary tumor. The restrictive inclusion criterion used during the qualification of patients for the study will facilitate the proper selection of patients for adjuvant intravesical BCG immunotherapy, which is particularly important because of the potential adverse effects associated with the therapy. Moreover, this model is simple, feasible, and relatively inexpensive. Nevertheless, our study has several limitations, which include the low sample size, its retrospective and single-centered nature, and the lack of external validation. The model must be validated by other centers or through the future prospective studies. Nevertheless, the data from our study significantly improved the state of knowledge about prognostic factors in patients with non*-*muscle-invasive bladder cancer. The obtained results also provide new prognostic tools that can be successfully applied in everyday clinical practice.
